# A comparison of brief versus explicit descriptors for verbal rating scales: interrupted time series design

**DOI:** 10.1186/s12955-023-02184-0

**Published:** 2023-09-13

**Authors:** Andrew J. Vickers, Melissa Assel, Michael Hannon, Priyanka Desai, Sigrid V. Carlsson, Taylor McCready, Jennifer Cracchiolo, Brett Simon

**Affiliations:** 1 Departments of Epidemiology and Biostatistics (AV, MA, SC), Anesthesiology (PD, TM, BS) and Surgery (MH, JC, SC), Memorial Sloan Kettering Cancer Center, 485 Lexington Ave, 2Nd Floor, New York, NY 10017 USA; 2 Department of Urology, (SC) Sahlgrenska Academy at Gothenburg University, Gothenburg, Sweden

**Keywords:** Patient reported outcomes, Comparative study, Psychometrics, Validation study, Postoperative period

## Abstract

**Background:**

Verbal rating scales (VRS) are widely used in patient-reported outcome (PRO) measures. At our institution, patients complete an online instrument using VRSs with a five-point brief response scale to assess symptoms as part of routine follow-up after ambulatory cancer surgery. We received feedback from patients that the brief VRS descriptors such as “mild” or “somewhat” were vague. We added explicit descriptors to our VRSs, for instance, “Mild: I can generally ignore my pain” for pain severity or “Somewhat: I can do some things okay, but most of my daily activities are harder because of fatigue” for fatigue interference. We then compared responses before and after this change was made.

**Methods:**

The symptoms investigated were pain, fatigue and nausea. Our hypothesis was that the explicit descriptors would reduce overall variance. We therefore compared the coefficient of variation of scores and tested the association between symptoms scores and known predictors thereof. We also compared time to completion between questionnaires with and without the additional descriptors.

**Results:**

A total of 17,500 patients undergoing 21,497 operations were assigned questionnaires in the period before the descriptors were added; allowing for a short transition period, 1,417 patients having 1436 operations were assigned questionnaires with the additional descriptors. Symptom scores were about 10% lower with the additional descriptors but the coefficient of variation was slightly higher. Moreover, the only statistically significant difference between groups for association with a known predictor favored the item without the additional language for nausea severity (*p* = 0.004). Total completion time was longer when the instrument included the additional descriptors, particularly the first and second time that the questionnaire was completed.

**Conclusions:**

Adding descriptors to a VRS of post-operative symptoms did not improve scale properties in patients undergoing ambulatory cancer surgery. We have removed the additional descriptors from our tool. We recommend further comparative psychometric research using data from PROs collected as part of routine clinical care.

**Supplementary Information:**

The online version contains supplementary material available at 10.1186/s12955-023-02184-0.

## Introduction

Verbal rating scales (VRS) are widely used in patient-reported outcome (PRO) measures. In a typical application, the patient is asked about symptom severity and given the response options “none / mild / moderate / severe” with or without the addition of a fifth option of “very severe”.

At Memorial Sloan Kettering Cancer Center (MSKCC) we use the five-item version of the VRS in our routine assessment of post-operative symptoms in patients undergoing ambulatory surgery [1]. Patients receive an online questionnaire called “Recovery Tracker”, every day for 10 days following surgery. Domains include pain, fatigue, nausea, vomiting, shortness of breath, constipation, swelling, bruising and wound discharge. The Recovery Tracker items are adapted from a validated symptom assessment instrument, the National Cancer Institute (NCI)’s Patient-Reported Outcomes version of the Common Terminology for Adverse Events (PRO-CTCAE) [2] The questionnaire is linked to an alerting system so that patients reporting, for instance, severe pain, are contacted by a nurse for follow-up [3]. We have demonstrated that use of the Recovery Tracker reduces avoidable urgent care visits and that patient anxiety is reduced when the Recovery Tracker is coupled with normative feedback to patients on how their symptoms compare to other similar patients [1].

We received informal feedback from patients that the VRS descriptors are vague. Patients told us that they were unsure how to interpret descriptors such as “mild” or “moderate”. A common occurrence was that a patient would report a symptom as “severe” on the Recovery Tracker, but would then be surprised when subsequently called by a nurse, stating that the symptom was perfectly manageable.

We had previously conducted research demonstrating the superiority of a VRS compared to a visual analog scale for post-exercise muscle soreness [4]. One feature of the VRS in that study was that it included explicit descriptors, for instance, “a light pain when walking up and down stairs” or “a light pain when walking on a flat surface”. We therefore considered whether adding language to the descriptors “mild”, “moderate” and “severe” would improve the properties of our VRS.

In a discussion amongst the clinical team, without the express input of patients, we chose to characterize symptom intensity in terms of mental intrusiveness. Using pain as an example symptom: “None or very mild: I have no pain or hardly any pain at all”; “Mild: I can generally ignore my pain”; “Moderate: I can ignore my pain at times”; “Severe: It is difficult to ignore my pain”; “Very severe: It is difficult to think about anything else”. Comparable language was used for other symptoms, for instance, “Moderate: I can ignore my fatigue at times”. For symptom interference, the additional descriptors were based on the degree of difficulty that the symptom caused for daily activities. Using pain interference as an example: “Not at all or very little: I was able to do my daily activities with very little trouble or no trouble at all”; “A little bit: I can do most of my daily activities without any problem, but some are a little harder because of pain”; “Somewhat: I can do some things okay, but most of my daily activities are harder because of pain”; “Quite a bit: Pain makes it hard to live my normal life”; “Very much: It is very difficult to do any of my daily activities because of my pain”. The full text of questions before and after the change is given in the Supplementary Material.

Rather than simply implementing the new VRS with explicit descriptors, we chose to study its characteristics in comparison to the VRS with the brief descriptors. We initially considered a randomized design but, given our large database of patients who had completed the VRS using simple descriptors, and the lack of any time trends in our data, we chose instead an interrupted time-series approach. We implemented the new descriptors and then compared the properties of the revised instrument to our historical experience. Our objective was to determine whether adding descriptors to a VRS measuring post-operative symptoms would improve its psychometric properties. These were defined in terms of the variance of symptom scores, and also the strength of the correlation between symptom scores and known predictors of post-operative outcomes.

## Methods

All patients in the study were undergoing ambulatory surgery for localized cancer at the Josie Robinson Surgery Center (JRSC) at MSKCC. The characteristics of patients treated at the JRSC have been described previously [1]. In general, patients need to be relatively young and healthy in order to qualify for short-stay cancer surgery. All patients at JRSC are offered participation in Recovery Tracker as a routine part of their clinical care. The Recovery Tracker went live at MSKCC on the following dates for the various services: 10/1/2016 in Urology, 4/15/2017 in Breast and Plastics, 6/12/2017 in Gynecology, and 12/11/2017 in Head and Neck. Questionnaires sent on or after November 10, 2021 included the additional descriptors.

Under a waiver from the Internal Review Board at MSK for retrospective research, we pulled data for all patients treated at JRSC from the date of the initiation of the Recovery Tracker in the respective services through February 21, 2022 to obtain just over 3 months of questionnaires that included the additional descriptors. We excluded patients who underwent surgery from October 30, 2021 to November 8, 2021 as they would have been transitioned between questionnaire types during the ten-day postoperative period when they receive the Recovery Tracker. We decided to analyze only pain, fatigue and nausea, on the grounds that other symptoms were rarely reported: in the case of shortness of breath, for instance, only 2% of responses indicated moderate or greater severity.

We hypothesized that adding descriptors to the items would decrease variance. As an illustration, take two patients who have very similar subjective experiences of pain on two consecutive days. It might be that they would nonetheless give different answers, one responding “mild” and the other “moderate” due to the vagueness of these terms; they might be less likely to give different responses for “generally ignore my pain” vs. “I can ignore my pain at times”. If the use of additional descriptors reduces this source of variance, that would reduce total variance and increase the correlation between symptom scores and known predictors of that symptom.

To test the former, we calculated the mean and standard deviation of each symptom, comparing between responses given with and without the additional descriptors. For the second hypothesis, we investigated predictors that have been established in the literature to be associated with each symptom: age with pain [5] and fatigue [6]; procedure type with pain; Apfel score and gender with nausea [7]; American Society of Anesthesiology (ASA) score and Body Mass Index (BMI) [6] with fatigue. To account for potential differences in types of procedures or patient characteristics among patients receiving questionnaires with and without additional descriptors, we split the latter into a training and test set. To establish predictors of each questionnaire question we randomly selected 2/3^rds^ of the operations from the initial period as our training dataset to test the association between predictors of interest and responses using multivariable mixed effects linear regression, adjusting for postoperative day (POD) of the questionnaire along with its cubic splines. As patients may have multiple surgeries and questionnaires are sent out PODs 1–10, we included a nested random effect intercept varying among patients and among surgeries within each patient. We selected statistically significant predictors established in the training set and use those predictors to build multivariable mixed effects linear regression models adjusting for POD (and cubic splines) separately in the remaining 1/3^rd^ of operations in the initial period and all responses using the additional descriptors, with a nested random effect intercept varying among patients and among surgeries within each patient. To test whether the effect of the selected predictors differed by cohort we tested for an interaction between age and questionnaires, with and without the additional descriptors, after adjusting for the main effects plus postoperative day (plus cubic splines), with the nested random effect using multivariable linear mixed effects models.

As a secondary analysis we tested whether there was a difference in the time to questionnaire completion by questionnaire type using a linear mixed effects model with time to questionnaire completion as the outcome. We used a nested random effect intercept varying among patients and among surgeries within each patient and a fixed effect for questionnaire type as the predictor of interest adjusting for number of prior questionnaires completed after their most recent surgery along with its cubic splines. All analyses were conducted using R 4.1.2.

## Results

A total of 21,497 operations for 17,500 patients were assigned questionnaires in the initial study period and 1,436 surgeries representing 1,417 patients were assigned questionnaires with additional descriptors. Patient characteristics by questionnaire type assigned are displayed in Table 1. There were some statistically significant differences between periods due to secular trends, but the absolute size of differences was very small, such as a difference in median age of 1 year and median operative time of 10 min.

**Table 1:** Patient characteristics by questionnaire period. Values are displayed as Median (IQR) or n (%). *P*-values were calculated using mixed effects models testing the association between questionnaire period as the predictor and the characteristic as the outcome with a random effect for patient

Characteristic	Initial period: brief descriptors N = 21,497	With explicit descriptors N = 1,436	*p*-value
Age	55 (46, 64)	56 (45, 64)	< 0.001
BMI	27 (23, 31)	27 (24, 31)	0.3
Unknown	3	0	
Female	16,724 (78%)	1,144 (80%)	0.7
ASA score (3–4)	8,358 (39%)	577 (40%)	0.4
Unknown	5	2	
Apfel Score			
0–2	4,944 (24%)	337 (23%)	0.005*
3	11,447 (55%)	840 (58%)	
4	4,471 (21%)	259 (18%)	
Unknown	635	0	
Service			
Breast	5,885 (27%)	368 (26%)	< 0.001*
Gynecology	3,461 (16%)	303 (21%)	
Head and Neck	1,344 (6.3%)	115 (8.0%)	
Plastics	6,482 (30%)	402 (28%)	
Urology	4,325 (20%)	248 (17%)	
Outpatient Surgery	9,054 (42%)	600 (42%)	0.8
Operative time (mins)	122 (74, 200)	112 (67, 180)	< 0.001

*BMI* Body Mass Index, *ASA* American Society of Anesthesiology

^*^p-values calculated where questionnaire period is the outcome and characteristic is the predictor

Symptom scores in each period are shown in Table 2. Use of the additional descriptors led to about a 10% decrease in scores. The key result was in the opposite direction to our hypothesis, the coefficient of variation was generally higher during the period when patients receiving items with additional descriptors.

**Table 2:** Postoperative symptom severity and interference by type of instrument received among patients in the test set. Values are displayed as mean (standard deviation) [coefficient of variation] combining all postoperative days. Differences and 95% confidence intervals represent the decrease in mean score among those who received questionnaires with additional descriptors calculated using linear mixed effects regression model with the questionnaire score as the outcome with a nested random effect intercept varying among patients and among surgeries within each patient. The N’s are the number of surveys

Characteristic	Initial period: brief descriptors, *N* = 66,962	With explicit descriptors, *N* = 13,544	Difference (95% Confidence Interval)
Pain Severity	2.19 (0.77) [0.35]	2.02 (0.85) [0.42]	0.16 (0.12, 0.20)
Not answered	35,893	5,692	
Fatigue Severity	2.04 (0.78) [0.38]	1.84 (0.85) [0.46]	0.21 (0.17, 0.25)
Not answered	35,856	5,690	
Nausea Severity	1.27 (0.60) [0.47]	1.11 (0.39) [0.36]	0.19 (0.16, 0.22)
Not answered	35,884	5,686	
Pain Interference	2.36 (0.95) [0.40]	2.09 (0.92) [0.44]	0.25 (0.20, 0.30)
Not answered	41,092	7,288	
Fatigue Interference	2.40 (0.91) [0.38]	2.19 (0.85) [0.39]	0.26 (0.21, 0.31)
Not answered	43,424	9,031	

For our analyses studying the association between predictors and symptom scores, the results of the preliminary analyses on the training set are shown in Supplemental Tables 1– 5. We found that age was a strong predictor of symptom responses. We therefore compared age coefficient estimates generated from separate multivariable linear mixed effects models in the two time periods separately for each item. The results are shown in Table 3. We did not find evidence of a difference in the size of the association (*p* ≥ 0.1) except for nausea severity, where there was a statistically significant difference in the opposite direction to that hypothesized (*p* = 0.020). In an additional analysis for the nausea symptom, the size of the association between Apfel score and outcome was also significantly smaller when the additional descriptors were used (*p* = 0.006).

**Table 3:** Association between age and responses among patients in the test set. Values represent the coefficient and standard error generated from multivariable linear mixed effects models testing the association between age (per 20-year increase) and questionnaire responses after adjusting for postoperative day (plus cubic splines) with a nested random effect intercept varying among patients and among surgeries within each patient separately in those receiving additional descriptors and the test set of those receiving questionnaires without additional descriptors. The *p*-value is that for an interaction term between age and type of descriptor

Outcome	Brief Descriptors Age Coefficient (SE)	Explicit Descriptors Age Coefficient (SE)	*p*-value
Pain Severity	-0.131 (0.014)	-0.136 (0.031)	0.8
Fatigue Severity	-0.066 (0.015)	-0.115 (0.032)	0.12
Nausea Severity	-0.097 (0.012)	-0.046 (0.014)	0.020
Pain Interference	-0.164 (0.019)	-0.165 (0.037)	> 0.9
Fatigue Interference	-0.072 (0.019)	-0.048 (0.036)	0.5

**Fig. 1:**
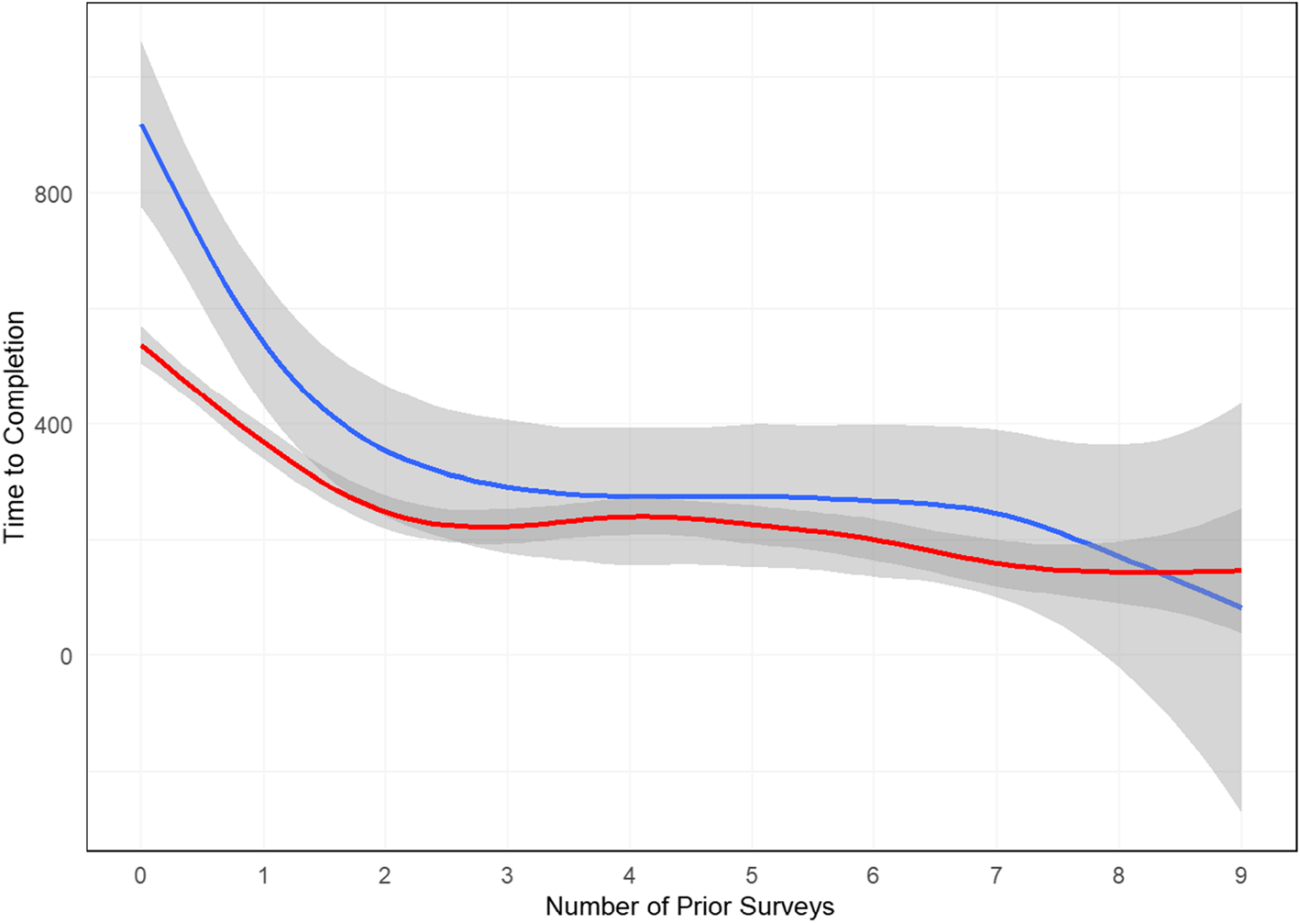
Generalized additive model estimating time to questionnaire completion by the number of prior questionnaires completed. The blue line represents questionnaires with the additional descriptors and the red line without the additional descriptors. Shaded areas represent 95% confidence intervals

Mean time to questionnaire completion is displayed in Fig. 1. The interaction between type of questionnaire and number of prior questionnaires seen was significant (*p* < 0.001). It took patients much longer to complete the questionnaire with additional descriptors for the first and second questionnaire seen; the estimated time to completion was 16 vs 9.1 min and 9.2 vs 6.1 min. The time to completion was relatively similar thereafter.

## Discussion

We hypothesized that adding explicit descriptors to a VRS used in a PRO instrument would decrease the variance of PRO scores and improve correlation with known predictors, without unduly affecting time to questionnaire completion. We found, instead, that use of the additional descriptors increased time to completion importantly, particularly for the first questionnaires completed by the patient, without any beneficial effects on PRO properties. We have accordingly switched back to simple descriptors without the additional language for use in clinical practice. We did so even though the instrument with the additional descriptors would no doubt meet the typical criteria for validation of a PRO instrument.

Our study is a rare example of health-related, psychometric research comparing two versions of the same item. It is not unusual for entire instruments to be compared. For instance, Hjermstad et al. reported a systematic review of 54 papers comparing VRS, visual analog scales (VAS) and numerical rating scales (NRS) of pain [8]. Another common approach is to see whether a shorter version of a questionnaire can be used in place of a longer one. El-Baalbaki et al., for instance, compared the 15-item short-form McGill Pain Questionnaire (MPQ-SF) to a single item NRS pain measure in patients with systemic sclerosis. They concluded that there was not much advantage to the MPQ-SF and that the NRS should be used instead due to its lower patient burden [9]. A similar type of study is where fixed questionnaires are compared to those with computer-adaptive testing [10]. Studies have also compared modes of administration – electronic versus paper [11] or interview versus self-administration [12] – or different recall periods – for instance, shorter versus up to 4 week recall periods are generally comparable for fatigue [13], urinary function [14] or physical functioning [15].

That said, there are few quantitative analyses comparing versions of the same health-related questionnaire with alternative wording choices. Most typically, a questionnaire is developed, from initial focus groups with patients to external validation, with quantitative comparison restricted to item selection. To illustrate this point, we chose, pretty much at random, the Anaphylaxis Quality of Life Scale for Adults [16]. The investigators interviewed some patients newly diagnosed with anaphylaxis and analyzed the transcripts for themes. Following further discussion with psychologists and allergy specialists, the investigators developed a 28-item prototype scale with five response options: never / rarely / sometimes / most of the time / always. This was administered to 115 participants, with factor analysis used to create three domains (social, emotional, limitations) and to remove seven items that did not correlate well with other items. The investigators found that the resulting scale correlated well with other measures of quality of life and recommended its use for research and clinical practice. However, at no point did the authors quantitatively compare different wordings. For instance, the item “Having anaphylaxis stops me getting on with my life” is included in the scale because it correlated reasonably well with other items, not because it was demonstrated to be superior to alternatives such as, say, “I feel I cannot plan for the future because of my anaphylaxis” or “Because of my anaphylaxis, my life isn’t where it should be”. Similarly, the response options “never / rarely / sometimes / most of the time / always” were never compared with alternatives such as “strongly agree / agree / neutral / disagree / strongly disagree”.

Of interest, in their review of pain instruments [8], Hjermstad et al. explicitly recommend this sort of research: “Whether the variability in anchors and response options directly influences the numerical scores needs to be empirically tested.” We have found only a few examples. Cook et al. undertook a modeling study suggesting that two or three response options on a NRS was too few, 5 was adequate and 11 unlikely to be additional benefit [17]. Similar findings have been reported in the general psychometric literature, for example, for personality assessment scales [18].

Our experience demonstrates that comparative research on PROs can be conducted easily and inexpensively when piggy-backed on electronic PROs implemented as part of routine clinical care. We were able to analyze data on over 50,000 questionnaires with zero costs for research data collection. The cost of the research is minor, being restricted to investigator meetings, regulatory administration (for the IRB waiver) and statistical analysis.

The size of our study is in some contrast with prospective research specifically conducted to investigate psychometric questions, which rarely includes more than 1000 respondents. This can have substantial implications for methodologic research. Take a study where patients received one of two different scales. To detect a 0.05 standard deviation (SD) difference between the scales would require ~ 12,500 subjects for 80% power. This is far from a trivial difference: a trial of a novel treatment with 80% power to detect a moderate effect size of 0.3 SD would have power of only 65% if using an inferior scale that resulted in a 0.25 SD difference between groups.

A possible limitation of our study is the relatively high rate of unanswered items. This is expected as, first, not all patients have access to the patient portal and second, many patients stop answering the daily questionnaires before the final one is sent at 10 days because they have fully recovered and are not experiencing any operative symptoms at that point. The rate of missing data is slightly lower for the additional descriptors, likely due to increased use of the portal over time. However, there is no reasonable mechanism by which missing data could have an important effect on our main finding that use of additional descriptors did not improve the association between symptom scores and known predictors thereof.

While we have removed the additional descriptors for the Recovery Tracker, we would caution against any over-interpretation of our findings. It would be unsound to make a general conclusion that additional descriptors for symptom states are unhelpful. First, the value of additional descriptors may depend on mode of administration. Specifically, about 70% of responses were completed using a mobile phone, where the small screen would favor a shorter response option. Second, additional descriptors may have greater or lesser utility depending on chronicity or type of symptom. For instance, the additional descriptors were particularly problematic for nausea. This may be because symptom tends to come and go during the course of a day, compared to pain, which is a more constant level of severity. Indeed, the poorer properties of the item with additional descriptors may be related to a focus on severity rather than duration: the original item was “how often do you have nausea?”. Third, there may be better descriptors for severity than those based on the mental intrusiveness of a symptom, and better descriptors for interference than those based on difficulty with everyday activities. One obvious explanation for our findings is that the additional descriptors led to additional variation, for instance, patients varied in how they interpreted “generally ignore” compared to “ignore at times”. Hence further research might examine alternative descriptors less open to variations in interpretation. Research might also examine whether additional descriptors might be of value in situations where patients experience only one symptom at a time, as it is plausible that perceptions of how much a symptom can be ignored depend on the presence of other symptoms.

In conclusion, adding descriptors to a verbal rating scale of post-operative symptoms did not improve scale properties in patients undergoing ambulatory cancer surgery. We recommend further comparative psychometric research using data from PROs collected as part of routine clinical care.

### Supplementary Information

**Additional file 1.**

## Data Availability

Manuscript has data included as electronic supplementary material.
