# Supplementary material for: A comparison of brief versus explicit descriptors for verbal rating scales: interrupted time series design
Source: Health Qual Life Outcomes. 2023 Sep 13;21:105. doi: 10.1186/s12955-023-02184-0 (PMC10498613; doi:10.1186/s12955-023-02184-0)
Supplement: Supplementary file 1 — Additional file 1. [file 12955_2023_2184_MOESM1_ESM.docx]

**Supplementary Appendix**

Text of item responses with and without additional descriptors

| **Initial Items** | **With additional descriptors** |
| --- | --- |
| **Pain Severity**  What is the severity of your pain at its worst?   1. None 2. Mild 3. Moderate 4. Severe 5. Very severe | **Pain Severity**  How would you describe your pain?   1. None or very mild: I have no pain or hardly any pain at all 2. Mild: I can generally ignore my pain 3. Moderate: I can ignore my pain at times 4. Severe: It is difficult to ignore my pain 5. Very severe: It is difficult to think about anything else |
| **Pain Interference**  How much does pain interfere with your usual or daily activities?   1. Not at all 2. A little bit 3. Somewhat 4. Quite a bit 5. Very much | **Pain Interference**  How much did your pain make it hard to do your daily activities?   1. Not at all or very little: I was able to do my daily activities with very little trouble or no trouble at all 2. A little bit: I can do most of my daily activities without any problem, but some are a little harder because of pain 3. Somewhat: I can do some things okay, but most of my daily activities are harder because of pain 4. Quite a bit: Pain makes it hard to live my normal life 5. Very much: It is very difficult to do any of my daily activities because of my pain |
| **Nausea Frequency**  How often do you have nausea (feeling like you are going to throw up)?   1. Never 2. Rarely 3. Occasionally 4. Frequently 5. Almost Constantly | **Nausea Severity**  How would you describe your nausea (feeling like you are going to throw up)?   1. None or very mild: I have no nausea or hardly any nausea at all 2. Mild: I can generally ignore my nausea 3. Moderate: I can ignore my nausea at times 4. Severe: It is difficult to ignore my nausea 5. Very Severe: It is difficult to think about anything else |
| **Vomiting Frequency**  How often do you have vomiting?   1. Never 2. Rarely 3. Occasionally 4. Frequently 5. Almost Constantly | **Vomiting Frequency**  How much did you vomit (throw up) in the past 24 hours?   1. None or hardly any: I may have sometimes gagged a bit or felt vomit in my mouth 2. Rarely: I have vomited 1 to 2 times 3. Sometimes: I have vomited 3 to 5 times 4. Frequently: I have vomited 6 or more times 5. Almost constantly: I have been vomiting almost constantly |
| **Fatigue Severity**  What is the severity of your fatigue (feeling tired, having less energy than usual) at its worst?   1. None 2. Mild 3. Moderate 4. Severe 5. Very severe | **Fatigue Severity**  How would you describe your fatigue?   1. None or very mild: I have no fatigue or hardly any fatigue at all 2. Mild: I can generally ignore my fatigue 3. Moderate: I can ignore my fatigue at times 4. Severe: It is difficult to ignore my fatigue 5. Very severe: It is difficult to think about anything else |
| **Initial Items** | **With additional descriptors** |
| **Fatigue Interference**  How much did your fatigue (feeling tired, having less energy than usual) make it hard to do your usual activities?   1. Not at all 2. A little bit 3. Somewhat 4. Quite a bit 5. Very much | **Fatigue Interference**  How much did your fatigue make it hard to do your daily activities?   1. Not at all or very little: I was able to do my daily activities with very little trouble or no trouble at all 2. A little bit: I can do most of my daily activities without any problem, but some are a little harder because of fatigue 3. Somewhat: I can do some things okay, but most of my daily activities are harder because of fatigue 4. Quite a bit: Fatigue makes it hard to live my normal life 5. Very much: It is very difficult to do any of my daily activities because of my fatigue |
| **Shortness of Breath Severity**  What is the severity of your shortness of breath at its worst?   1. None 2. Mild 3. Moderate 4. Severe 5. Very severe | **Shortness of Breath Severity**  How would you describe your shortness of breath?   1. None or very mild: I have no shortness of breath or hardly any shortness of breath at all 2. Mild: I can generally ignore my shortness of breath 3. Moderate: I can ignore my shortness of breath at times 4. Severe: It is difficult to ignore my shortness of breath 5. Very severe: It is difficult to think about anything else |
| **Hoarseness Severity**  How would you describe your hoarseness (weak, breathy, scratchy, or raspy sound to your voice)?   1. None 2. Mild 3. Moderate 4. Severe 5. Very severe | **Hoarseness Severity**  How would you describe your hoarseness (weak, breathy, scratchy, or raspy sound to your voice)?   1. None or very mild: I have no hoarseness or hardly any hoarseness 2. Mild: I can generally ignore my hoarseness 3. Moderate: I can ignore my hoarseness at times 4. Severe: It is difficult to ignore my hoarseness 5. Very Severe: It is difficult to think about anything else |
| **Numbness or Tingling Severity**  How would you describe the numbness or tingling in your hands, feet, or around the mouth?   1. None 2. Mild 3. Moderate 4. Severe 5. Very severe | **Numbness or Tingling Severity**  How would you describe the numbness or tingling in your hands, feet, or around the mouth?   1. None or very mild: I have no or hardly any numbness or tingling 2. Mild: I can generally ignore my numbness or tingling 3. Moderate: I can ignore my numbness or tingling at times 4. Severe: It is difficult to ignore my numbness or tingling 5. Very Severe: It is difficult to think about anything else |

Supplemental Table 1. Separate multivariable linear regression models testing predictors of interest with pain severity adjusting for postoperative day (along with cubic splines) with a nested random effect intercept varying among patients and among surgeries within each patient in the training set.

| **Characteristic** | **N** | **β** | **95% Confidence Interval** | **p-value** |
| --- | --- | --- | --- | --- |
| Age (per 10 year increase) | 62,190 | -0.06 | -0.07, -0.05 | <0.001 |
| Mastectomy w/TE vs no TE | 13,463 | 0.32 | 0.26, 0.38 | <0.001 |
| Bilateral Mastectomy w/TE vs Unilateral | 10,134 | 0.21 | 0.15, 0.27 | <0.001 |

TE=Tissue Expander

Supplemental Table 2: Separate multivariable linear regression models testing predictors of interest with pain interference adjusting for postoperative day (along with cubic splines) with a nested random effect intercept varying among patients and among surgeries within each patient in the training set.

| **Characteristic** | **N** | **β** | **95% Confidence Interval** | **p-value** |
| --- | --- | --- | --- | --- |
| Age (per 10 year increase) | 51,454 | -0.08 | -0.09, -0.06 | <0.001 |
| Mastectomy w/TE vs no TE | 12,003 | 0.30 | 0.22, 0.38 | <0.001 |
| Bilateral Mastectomy w/TE vs Unilateral | 9,276 | 0.30 | 0.22, 0.37 | <0.001 |

Supplemental Table 3: Separate multivariable linear regression models testing predictors of interest with fatigue severity adjusting for postoperative day (along with cubic splines) with a nested random effect intercept varying among patients and among surgeries within each patient in the training set.

| **Characteristic** | **N** | **β** | **95% Confidence Interval** | **p-value** |
| --- | --- | --- | --- | --- |
| ASA (3-4) | 62,228 | 0.03 | 0.01, 0.06 | 0.011 |
| Age (per 10 year increase) | 62,245 | -0.02 | -0.03, -0.01 | <0.001 |
| BMI (per 10 unit increase) | 62,239 | -0.01 | -0.03, 0.01 | 0.3 |

Supplemental Table 4: Separate multivariable linear regression models testing predictors of interest with fatigue interference adjusting for postoperative day (along with cubic splines) with a nested random effect intercept varying among patients and among surgeries within each patient in the training set.

| **Characteristic** | **N** | **β** | **95% Confidence Interval** | **p-value** |
| --- | --- | --- | --- | --- |
| ASA (3-4) | 47,375 | 0.02 | -0.01, 0.05 | 0.3 |
| Age (per 10 year increase) | 47,392 | -0.03 | -0.04, -0.01 | <0.001 |
| BMI (per 10 unit increase) | 47,387 | -0.03 | -0.05, 0.00 | 0.045 |

Supplemental Table 5: Separate multivariable linear regression models testing predictors of interest with nausea severity adjusting for postoperative day (along with cubic splines) with a nested random effect intercept varying among patients and among surgeries within each patient in the training set.

| **Characteristic** | **N** | **β** | **95% Confidence Interval** | **p-value** |
| --- | --- | --- | --- | --- |
| Female | 62,205 | 0.18 | 0.16, 0.20 | <0.001 |
| Apfel Score | 61,039 | 0.10 | 0.09, 0.12 | <0.001 |
